# Standardised survival probabilities: a useful and informative tool for reporting regression models for survival data

**DOI:** 10.1038/s41416-022-01949-6

**Published:** 2022-09-01

**Authors:** Elisavet Syriopoulou, Tove Wästerlid, Paul C. Lambert, Therese M.-L. Andersson

**Affiliations:** 1 Department of Medical Epidemiology and Biostatistics, Karolinska Institutet, Stockholm, Sweden; 2 Clinical Epidemiology Division, Department of Medicine Solna, Karolinska Institutet, Stockholm, Sweden; 3 Department of Hematology, Karolinska University Hospital, Stockholm, Sweden; 4 Biostatistics Research Group, Department of Health Sciences, University of Leicester, Leicester, UK

**Keywords:** Cancer epidemiology, Cancer models

## Abstract

**Background:**

When interested in studying the effect of a treatment (or other exposure) on a time-to-event outcome, the most popular approach is to estimate survival probabilities using the Kaplan–Meier estimator. In the presence of confounding, regression models are fitted, and results are often summarised as hazard ratios. However, despite their broad use, hazard ratios are frequently misinterpreted as relative risks instead of relative rates.

**Methods:**

We discuss measures for summarising the analysis from a regression model that overcome some of the limitations associated with hazard ratios. Such measures are the standardised survival probabilities for treated and untreated: survival probabilities if everyone in the population received treatment and if everyone did not. The difference between treatment arms can be calculated to provide a measure for the treatment effect.

**Results:**

Using publicly available data on breast cancer, we demonstrated the usefulness of standardised survival probabilities for comparing the experience between treated and untreated after adjusting for confounding. We also showed that additional important research questions can be addressed by standardising among subgroups of the total population.

**Discussion:**

Standardised survival probabilities are a useful way to report the treatment effect while adjusting for confounding and have an informative interpretation in terms of risk.

## Background

When analysing time-to-event data (or survival data) from epidemiological cohort studies in which a specific treatment (or exposure) is under study, it is often of interest to compare survival probabilities between treated and untreated patients. It is important to note that the term survival probabilities does not necessarily refer to being alive or not. Instead, it refers to being event-free. For instance, when time to relapse or death (whichever occurred first) is under study, survival probabilities refer to the probability of being alive without having a relapse (this is often referred to as relapse-free survival). Survival probabilities can also be interpreted as the proportion of individuals who are event-free at this time. When there are competing events, the survival probabilities can under some assumptions be interpreted as the probability of being event-free (or the proportion of individuals who are event-free) in the absence of competing events. We will not discuss these assumptions here as they are out of the scope of this paper, but we instead refer the reader to other related literature [1]. Survival probabilities can be compared using the Kaplan–Meier estimator [2]. However, since confounding variables might drive part of the observed differences in survival probabilities, researchers often adjust for these potential confounder variables by fitting regression models, such as the Cox proportional hazards model [3, 4]. A common practice after fitting regression models is to summarise differences between treatment or exposure groups using adjusted hazard ratios. The hazard ratio for treatment is defined as the ratio of the hazard rates for the treated and untreated.

Despite the popularity and broad use of hazard ratios, these are often misinterpreted as relative risks. Several authors have stressed the difference between hazard ratios and relative risks previously, but their interpretation remains loose to this day [5–8]. A measure that has a more intuitive and easier interpretation than hazard ratios and may be more relevant in several applications is the survival probability. The survival probability at a specific time is the probability that an individual did not have the event until that specified time. The Kaplan–Meier method is a crude, i.e. unadjusted, measure of the survival probability at a specified time. The survival probabilities under different treatment arms or exposure groups can be compared by calculating their difference and can provide a measure for estimating the association between treatment (or other exposure) and a specified outcome. An appealing feature of using survival probabilities is that their complement (1 minus survival probabilities) can be interpreted as the risk of experiencing the event by a specific time, which is often the quantity of interest. In fact, the popularity of Kaplan–Meier estimates as a crude measure in various studies, even though the potential for confounding is known, highlights the importance of presenting data in terms of survival probabilities as this is easier to interpret than hazard rates.

However, there are methods to obtain survival curves that are similar to Kaplan–Meier estimates, but adjusted for potential confounders. After fitting a regression model including various confounding variables, estimates of adjusted survival probabilities by treatment arms can be obtained and presented graphically. These are called adjusted survival curves and are similar to a Kaplan–Meier curve but have been adjusted for potential confounders. Typically, after fitting a regression model, adjusted survival curves by treatment status are estimated by setting all other variables to the mean value of the population [9]. However, the average values of confounding variables may be implausible and with no useful meaning for the cohort in the study. Instead, a more appropriate way to graphically report regression models may be to estimate adjusted survival curves using standardisation (so-called standardised survival curves). To do this, the individual-specific survival probabilities across all patients in the study population are estimated and averaged to obtain the standardised survival estimates (i.e. by applying regression standardisation) [10, 11]. In this way, we obtain estimates of the survival probabilities under the observed covariate pattern of the overall study population.

In this paper, we will discuss how we can summarise and present the analysis of a regression model in a useful way using standardised survival probabilities. This will be presented using plain language and with a practical focus. We will use publicly available data on breast cancer patients to demonstrate the different measures that can be estimated, including survival probabilities after fitting regression models with various confounders. We will also provide Stata code in the supplementary material to encourage the use of standardised survival probabilities in practice.

## Introducing the illustrative example

For the remainder of the paper, we will use an example on breast cancer to illustrate the concepts. This dataset has been used in several applications and is publicly available at http://www.stata-press.com/data/fpsaus.html [12, 13]. The dataset consists of 2982 primary breast cancer patients included in the Rotterdam tumour bank. The exposure of interest is hormonal therapy, and in the remainder of the paper, we will refer to a comparison of treatment groups even though these could also be exposure groups. The methods discussed are applicable to any other exposure that might be of interest (e.g., stage, deprivation status). The outcome of interest in our example is time to relapse or death (measured as time from primary surgery to relapse or death, whichever occurred first, and is also known as relapse-free survival). However, the methods discussed are applicable to any time-to-event outcome (e.g., overall survival, metastasis-free survival). Data include information on several factors, including age at surgery, tumour size, differentiation grade, number of positive nodes and progesterone level. More details on the data can be found in Table 1.

**Table 1:** Summary characteristics of breast cancer patients by treatment arm.

Variables	Hormonal therapy: no	Hormonal therapy: yes	Total
	*N* = 2643	*N* = 339	*N* = 2982
Age at surgery (years)	53 (44–64)	62 (57–69)	54 (45–65)
Number of positive nodes	0 (0–3)	4 (2–9)	1 (0–4)
Progesterone level (fmol/l)	46 (5–208)	19 (1–117)	41 (4–198)
Differentiation grade			
2	735 (28%)	59 (17%)	794 (27%)
3	1908 (72%)	280 (83%)	2188 (73%)
Tumour size			
<=20 mm	1283 (49%)	104 (31%)	1387 (47%)
>20–50 mm	1119 (42%)	172 (51%)	1291 (43%)
>50 mm	241 (9%)	63 (18%)	304 (10%)

For categorical variables, the number of individuals with relevant proportions is given.

For continuous variables, median with 25th and 75th percentiles are given.

## Exploring differences in survival probabilities between treatment groups with the Kaplan–Meier estimator

**Fig. 1:**
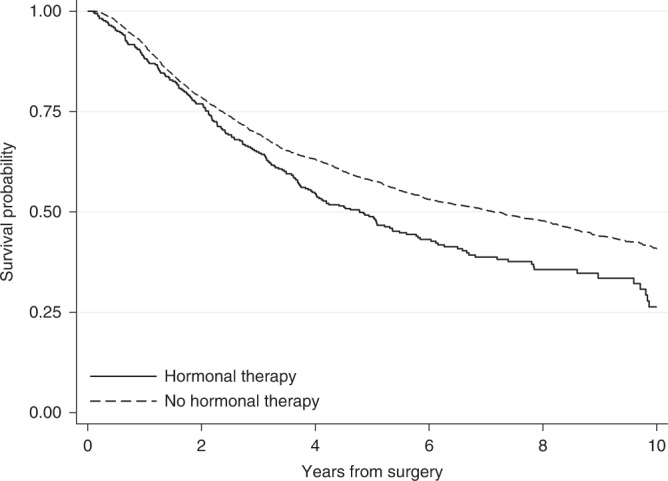
Kaplan–Meier survival curves. Kaplan–Meier survival probabilities for the event of relapse-free survival by treatment group for breast cancer patients.

To explore whether treatment affects a time-to-event outcome, the survival probabilities under different treatment arms are frequently compared using the Kaplan–Meier estimator [2, 3]. For instance, Fig. 1 shows the Kaplan–Meier survival probabilities for the outcome of relapse-free survival for the breast cancer patients who received hormonal therapy or not in the example dataset. The event of interest in this example is death or relapse (whichever occurs first). Here, survival probabilities are interpreted in terms of not only being alive but also having no relapse.

Based on the Kaplan–Meier curves, hormonal therapy seems to have an adverse effect on breast cancer patients. Ten years after surgery, the survival probability for relapse-free survival (i.e. the probability of being alive with no relapse) for those who received hormonal therapy is 0.26, while those who did not receive hormonal therapy had a higher probability of 0.41. This can also be interpreted in terms of proportions: 26% of those who received hormonal therapy and 41% of those who did not receive hormonal therapy were alive with no relapse 10 years after surgery. However, these crude Kaplan–Meier estimates do not adjust for the fact that patients who received hormonal therapy were older (median age of 62 years versus 53 years in the no hormonal therapy group), had a higher number of positive nodes and that there was a larger proportion of patients with a tumour above 50 mm among those who received hormonal therapy (Table 1). These imbalances between treatment groups might drive part of the differences in probabilities of relapse-free survival and so it is important to consider these factors in the analysis.

Adjusting for multiple covariates at once can be done by estimating the Kaplan–Meier curves within subgroups of the population. If many potential factors are of interest this would require calculation of multiple Kaplan–Meier estimates, and for continuous covariates it would be possible only after categorisation. When there are several confounding variables, it may not be feasible to estimate Kaplan–Meier estimates within each subgroup, due to potentially low number of individuals within each strata. Also, it becomes difficult to summarise the results when the survival probability within each strata has to be reported. An additional limitation is that although there are ways to evaluate whether differences in the two survival curves are statistically significant (e.g using the log-rank test), an estimate of the magnitude of the difference between the groups is not provided [14]. An alternative and more popular way to explore differences in survival probabilities between treatment groups, while accounting for several covariates, is to fit regression models.

## Estimating hazard ratios using regression models

The most commonly applied statistical regression model when studying time-to-event outcomes is Cox’s proportional hazards model [15]. Other regression models are also available with some (e.g. so-called flexible parametric survival models) having advantages in terms of modelling time-dependent effects (i.e. relaxing the assumption of proportional hazards) and also in terms of predictions [12]. For the analysis of the breast cancer data, we will use flexible parametric survival models. However, in principle, any survival model could be fitted, including the Cox model. As the main focus of this paper is to describe different ways of summarising the results of a regression model and not the model itself, we skip details on flexible parametric survival models but more information on these can be found elsewhere [12, 16].

After fitting a survival model the analysis is often summarised using the hazard ratio (HR) which is the ratio of the event rates in the two groups we want to compare. The hazard rate of a particular group is the rate of individuals who experience the event under study over a short period of time, provided that the individuals have not experienced the event yet. For instance, by fitting a flexible parametric survival model to the breast cancer data including only hormonal therapy in the model, we obtain a HR equal to 1.33 (95% CI: 1.15–1.54). This is in agreement with the findings of the Kaplan–Meier estimator about an adverse effect of hormonal therapy, but also provides an estimate of the size of the difference between treatment groups, i.e. the hazard rate of patients who received hormonal therapy is 33% higher than the hazard rate of those who did not receive hormonal therapy. However, this model does not take into consideration the imbalances we see between treatment groups in Table 1. After fitting a flexible parametric model for hormonal therapy adjusting for age at surgery, the number of positive nodes, progesterone level, differentiation grade and tumour size and relaxing the proportionality assumption for tumour grade and the number of positive nodes, we obtain a HR suggestive of a protective treatment effect and equal to 0.76 (95% CI: 0.65–0.89) i.e. the hazard rate of those who received hormonal therapy is 24% (=1–0.76) lower than the hazard rate of those who did not receive hormonal therapy.

**Fig. 2:**
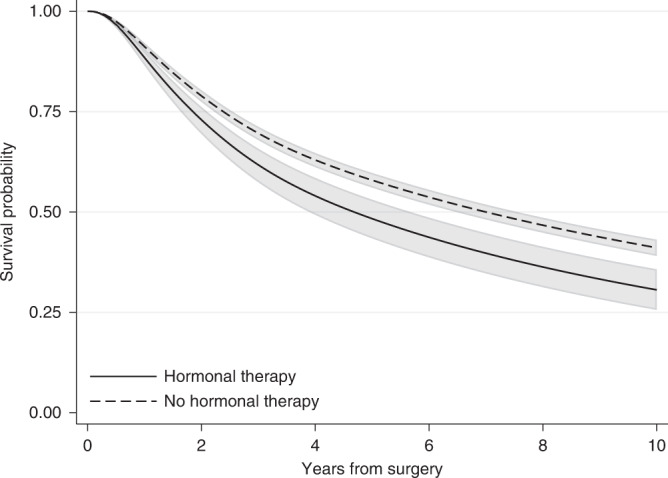
Survival probabilities for the event of relapse-free survival by treatment group for breast cancer patients, with 95% confidence intervals. Estimates are obtained after fitting a flexible parametric survival model including only hormonal therapy.

Many authors have previously argued about limitations related to the use of HRs. Despite the wide use of HRs to estimate the treatment effect, the interpretation of HRs remains challenging as HRs are often misinterpreted as relative risks [5, 7]. By definition the HR compares the rate of experiencing the event among treated and the rate of experiencing the event among untreated. This is different from the relative risk of experiencing the event by a specific time. The relative risk is the ratio of the probability of experiencing the event by a specific time for the treated to the probability for the untreated. In contrast to HR, the relative risk is always a function of time. For instance, the HR estimated for breast cancer patients after fitting a flexible parametric model that included only hormonal therapy and no confounding variables (unadjusted) was equal to 1.33 and remained constant during follow-up. However, the relative risk of having the event (relapse or death) is different and varies with time. The risk for treated and untreated can be obtained as 1 minus the survival probability estimates of Fig. 2, which shows the survival probabilities estimated from the same flexible parametric model that includes only hormonal therapy. These are similar to the Kaplan–Meier curves of Fig. 1 but are obtained from a regression model. As we can see in Fig. 2, both survival probabilities are close to each other early on, with values close to 1 since not many patients had relapsed or died in either treatment group, i.e. the risk of experiencing the event is close to 0 and the relative risk is ~1. One year after surgery, the risk of having the event for those who received hormonal therapy is 0.11 (=1–0.89) and for those who did not receive hormonal therapy 0.09 (=1–0.91), resulting in a relative risk of 1.22 (=0.11/0.09). Similarly, ten years after surgery the risk is equal to 0.69 (=1–0.31) for those who received hormonal therapy and 0.59 (=1–0.41) for those who did not receive it, resulting in a relative risk equal to 1.17 (=0.69/0.59). If we had allowed more follow-up time, the relative risk would approach 1 again later on, as the survival curves would eventually reach 0 both for treated and untreated (as all deaths will be realised eventually). However, this would not be the case when the event of interest is death due to a specific cause or when the event of interest is not death. As we can see by this example, the relative risk is highly dependent on the time of interest and its value differed from the HR estimate. HRs should be interpreted as relative rates and not relative risks. However, it is important to note that even though the HR estimate will be different to the relative risk value, the direction of the hazard ratio will be the same as the direction of the relative risk if the proportional hazards assumption is valid [5]. For example, a HR below 1 that corresponds to a lower hazard rate under treatment is also suggestive of a lower relative risk under treatment.

Another challenge with HRs is that studies often report a single HR estimate for the whole study follow-up. This was also the case in our example above. Thus, we assumed that the HR remains constant over time i.e. proportional hazards for treatment. However, this is often an unrealistic assumption and the HR will vary over time [17]. For instance, the effect of age might vary with time or the effect of a treatment may lose effectiveness over time. Thus, when the proportionality assumption is not valid, reporting a single hazard ratio is non-informative and can be very misleading. Several methods are available to obtain time-dependent HRs, but these are often overlooked. Moreover, the HR is a relative measure; although a HR lower than 1 suggests a protective effect of treatment and a HR higher than 1 suggest an adverse effect, HRs provide no information on the absolute effect or whether this effect is clinically meaningful. Statistically significant HRs indicate that there is a statistical significant difference between treatment groups, but the corresponding difference in survival probabilities might be very small and not important from a clinical point of view. Assessing the treatment effect by examining absolute measures such as the difference in survival probabilities at fixed time points can be more informative than relative measures [18]. Finally, HRs are estimated based on individuals who have survived up to a particular time. As time increases, the characteristics of individuals who are still in follow-up in each treatment group might differ, resulting in an imbalanced comparison between treatment groups. This is often referred to as built-in selection bias of hazard ratios [6, 19]. For instance, when an effective treatment is under study, as time increases there will be more patients with worse prognosis characteristics (e.g. older patients or patients with comorbidities) still alive among the treated group in comparison to the placebo group. This will be the case even if we have sufficiently adjusted for confounding and there are no imbalances between the treatment groups at the start of follow-up, due to emerging differences in characteristics between treated and untreated survivors with time. The selection bias of hazard ratios cannot be addressed even after appropriately modelling time-varying covariates and allowing for time-dependent covariate effects.

## Adjusted survival curves using the mean covariate method

**Fig. 3:**
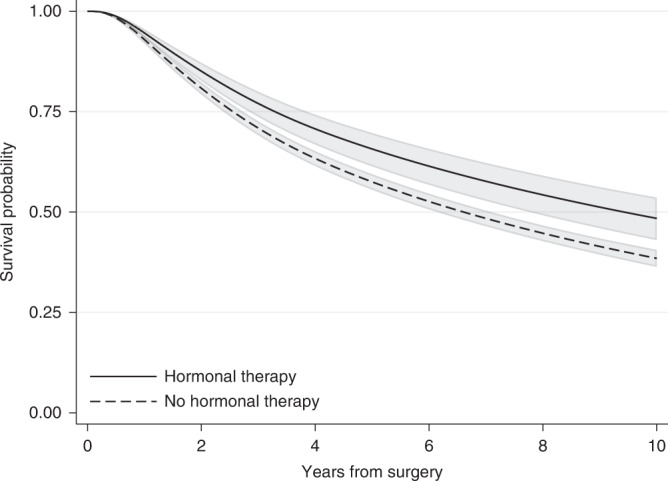
Adjusted survival probabilities for the event of relapse-free survival by treatment group for the “average” individual in the breast cancer study population. 95% confidence intervals are also provided.

A more informative way to summarise the treatment effect is to use adjusted survival probabilities. As mentioned earlier for the Kaplan–Meier estimator, the survival probability at a specific time corresponds to the probability of being event-free at a particular time after the beginning of follow-up (e.g., 5 years after surgery). Even though rarely reported, estimating survival probabilities after fitting a regression model is no more difficult than HRs and it can be obtained using standard statistical software. In a modelling context, when multiple covariates are included in the regression model, adjusted survival probabilities are often estimated using the average covariate value for the adjusting covariates. For the breast cancer example, after fitting the same flexible parametric survival model described earlier for the HR (with hormonal therapy as the treatment of interest and adjusting for various variables), 10 years after surgery the relapse-free survival probability (i.e. the probability of being alive with no relapse) was 0.48 (95% CI: 0.43–0.54) for those who received hormonal therapy and 0.39 (95% CI: 0.36–0.41) for those who did not receive it (Fig. 3). This can also be interpreted in terms of proportions: 48% of those who received hormonal therapy and 39% of those who did not receive hormonal therapy were alive without having a relapse 10 years after surgery. To obtain these estimates, we set all adjusting variables to their mean value, except the treatment that is first set to treated and then untreated, and an adjusted survival curve is estimated for each treatment arm. So, our estimates correspond to the survival probability of an “average” individual if this “average” individual received hormonal therapy and an “average” individual who did not receive hormonal therapy. Thus, a caveat with “naively” adjusted survival curves is the need to calculate an “average” for included variables. For continuous variables, such as age at surgery, the “average” individual in terms of the mean value might be easy to interpret. However, for categorical variables, such as tumour size, it is not clear what an average individual is [9]. The average value for a binary variable such as sex taking values 1 for females and 0 for males corresponds for instance to the proportion of individuals who were females (e.g. if 40% of the individual were females it will be equal to 0.4) and has no meaning on an individual level (as it does not correspond to either females or males). Also, for continuous variables, even though it is more straightforward to think about the mean value, this might still not be relevant to our study population. Imagine for example, a disease that is more common among individuals younger than 25 years old and older than 60 years old. In this example, the average age at diagnosis might not even correspond to a plausible patient profile. A way to overcome this is to obtain adjusted survival curves at fixed values for the adjusting variables but in this way the survival curves will still be restricted to a specific covariate pattern.

## Standardised survival curves

Another way to overcome the need to estimate adjusted survival probabilities for an “average” individual but still obtain adjusted survival probabilities that can be presented graphically is to apply regression standardisation and thus obtain so-called standardised survival curves (also known as marginal survival curves) [10, 11, 20, 21]. To do this, a regression model is fitted as in the previous examples. Based on this model, the standardised survival curve under treatment is obtained by first estimating individual-specific survival probabilities for every individual in the study population given the individual’s covariate pattern and if they received the treatment. It is important to note that “under treatment” is used here to be explicit about the fact that the covariate value used for the survival estimates for some individuals will be different from their observed value. We do not simply calculate an average over those who received treatment and an average over those who did not receive the treatment: this would result on comparing two groups with very different covariate distributions. For the standardised survival estimates under treatment, no change is made on the treatment covariate for those who were treated but the covariate value for those not treated is changed to treated. Then, the individual-specific survival probabilities are averaged to obtain the standardised survival probability under treatment. Similarly, the standardised survival curve under no treatment is estimated by averaging the individual-specific survival probabilities for each individual given the individuals covariate pattern but this time if everyone was untreated. For this, no change is made in the treatment covariate for those who were untreated but the covariate value for the treated is changed to untreated.

Note that for a study population of N individuals, N estimates of individual-specific survival probabilities are obtained and then averaged to obtain the standardised survival curve in the whole population under each treatment arm. This is different to the approach described in the previous section where only one survival curve is estimated for each treatment arm based on the average values of the adjusting covariates. With standardisation, instead of using an “average individual,” the empirical (i.e. observed) covariate distribution of the population is used for the estimate. Standardised survival estimates under treatment and no treatment can thus be interpreted as the average survival probabilities if everyone in the study population was treated or if everyone in the study population was untreated. Since the distribution of all other adjusting covariates are the same in the two standardised probabilities, fairer comparisons between treated and untreated can be made. An alternative interpretation for standardised survival estimates would be the proportion of individuals in the observed population that would survive if everyone was treated and the proportion of individuals in the observed population that would survive if no one was treated.

**Fig. 4:**
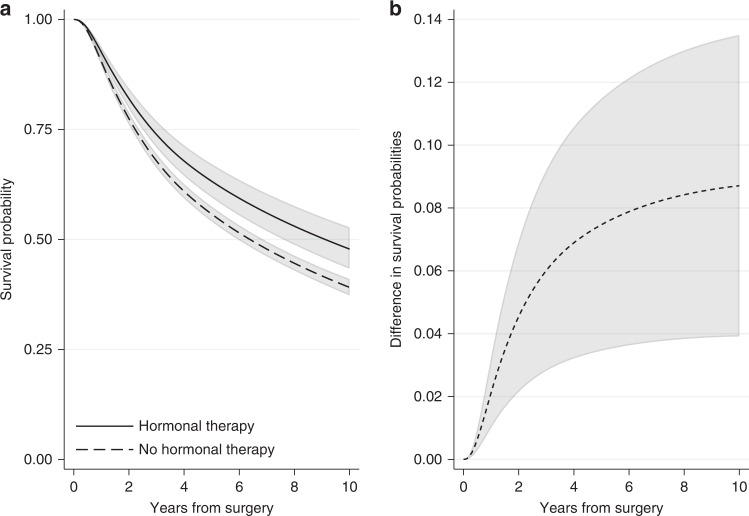
Standardised survival probabilities. **a** Standardised survival probabilities for the event of relapse-free survival by treatment group and **b** the difference in standardised survival probabilities for the event of relapse-free survival under hormonal therapy and under no hormonal therapy, with 95% confidence intervals. Standardisation is performed by using the empirical covariate distribution of the total breast cancer population.

In the breast cancer example, we can obtain the standardised survival curve under hormonal therapy as the average of the individual-specific survival probability estimates for the event of relapse-free survival if each individual received hormonal therapy over all individuals in the cohort. Similarly, the standardised survival curve under no hormonal therapy can be obtained by averaging across the individual-specific survival probability estimates if each individual did not receive hormonal therapy. Figure 4a shows the standardised survival curves under hormonal therapy and under no hormonal therapy. Ten years after surgery, the standardised survival probabilities are 0.48 (95% CI: 0.43–0.53) under hormonal therapy and 0.39 (95% CI: 0.37–0.41) under no hormonal therapy. Note that these standardised estimates are very close to the adjusted survival curves for an “average” individual (Fig. 3), but this will not always be the case. For instance, at 5 years since surgery, the standardised survival estimates are 0.63 (95% CI: 0.60–0.67) under hormonal therapy and 0.56 (95% CI: 0.54–0.58) under no hormonal therapy (Fig. 4a), while the adjusted survival probabilities for the “average” individual are higher: they are 0.66 (95% CI: 0.61–0.70) under hormonal therapy and 0.58 (95% CI: 0.56–0.59) under no hormonal therapy (Fig. 3). Estimates at 1, 5 and 10 years after surgery are also shown in Supplementary Table S1.

A comparison of the survival probabilities for different treatment groups can be performed by calculating the difference in the standardised survival probabilities under treatment and under no treatment. By applying the empirical covariate distribution in the survival estimates for both treatment groups, a fairer comparison between treated and not treated is obtained. The difference in standardised survival probabilities is a comparison of the probability of being event-free if all individuals had received treatment versus if they had not. An advantage of quantifying the treatment effect in terms of survival probabilities is that this can now be interpreted as risk (e.g., difference in risk of experiencing the event by a specific time under treatment in comparison to no treatment). Moreover, if the variables we had adjusted for are sufficient and there are no unmeasured confounders, the difference in standardised survival probabilities is an estimate of the causal effect of treatment on survival outcome [11]. This assumption is particularly important and its validity requires subject matter knowledge. Figure 4b shows the difference in standardised relapse-free survival probabilities under hormonal therapy and no hormonal therapy for the breast cancer population. The difference is increasing with time and ten years after surgery it is equal to 0.09 (95% CI: 0.04–0.14). Similarly, we can say that the proportion of being alive with no relapse under hormonal therapy is 9 percentage points larger than the proportion under no hormonal therapy.

**Fig. 5:**
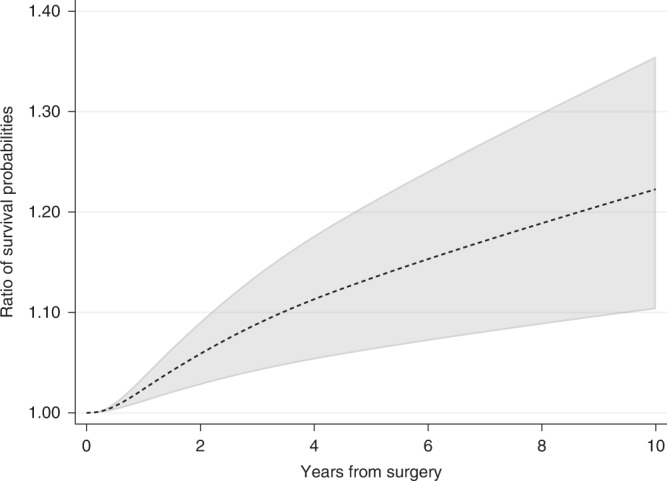
Ratio of the standardised survival probabilities for the event of relapse-free survival under hormonal therapy compared to the standardised survival probability under no hormonal therapy. 95% confidence intervals are also provided.

The ratio in survival probabilities can also be calculated if a relative measure is of interest. For instance, 10 years after surgery the ratio in survival probabilities for the event of relapse-free survival under hormonal therapy compared to no hormonal therapy is equal to 1.22 (95% CI: 1.10–1.36) (Fig. 5). However, absolute measures are often better for understanding if differences between groups are clinically meaningful. For example, if 60% of treated patients are event-free at 5 years compared to 40% for untreated, the absolute difference in proportion of event-free patients is 20 percentage points, equal to a ratio of proportions of 1.5 at 5 years. In a different study population, with a 5-year proportion of being event-free of 15% for treated and 10% for untreated, the ratio in proportions is also equal to 1.5, whereas the absolute difference is only 5 percentage points.

## Standardising within a subset of the study population

**Fig. 6:**
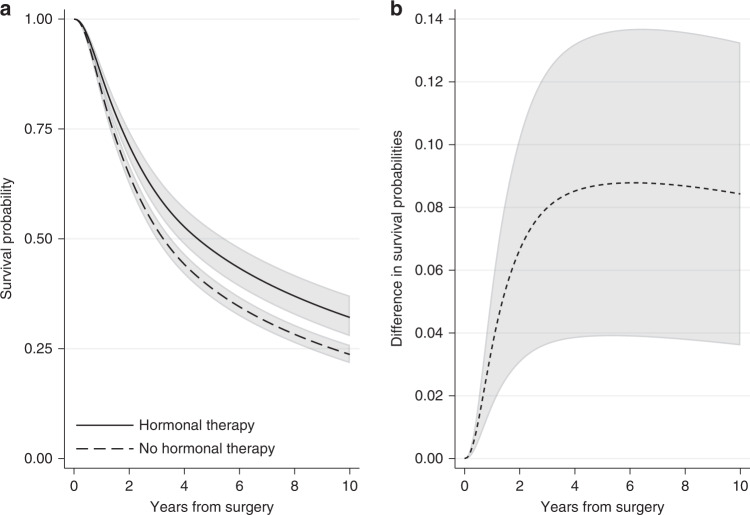
Standardised survival probabilities using the empirical covariate distribution of the breast cancer patients who received hormonal therapy. **a** Standardised survival probabilities for the event of relapse-free survival among patients who received treatment by treatment group and **b** the difference in standardised survival probabilities for the event of relapse-free survival under hormonal therapy and under no hormonal therapy, with 95% confidence intervals.

In the previous section, standardisation was performed using the empirical covariate distribution in the whole population, i.e., we estimated the average survival probability for the whole population if everyone was treated compared to if no one was treated. However, in some situations it may be more relevant to apply the empirical covariate distribution of a subset of the total study population, such as the covariate distribution among the treated. This would, for example, be the case when evaluating the impact of an intervention (e.g., a new treatment or a nutritional diet) in the population who actually received the intervention as opposed to evaluating the effect of the intervention in the total population (including patients to whom the intervention was never allocated). For instance, how large was the improvement in the probability of being alive with no relapse for the breast cancer patients who received hormonal therapy? Similarly, if the interest is to assess the potential impact of an intervention on a population who have not yet received it, it would be more relevant to apply the empirical distribution among the untreated. For instance, what would the improvement be in the probability of being alive and having no relapse if the untreated group had hormonal therapy? As was also the case when we standardised over the whole group, we need to assume that the treatment effect would be the same in this group (untreated). Such research questions are of high importance from a public health view and for policymakers. Providing estimates of the standardised survival probabilities within a subset of the total population is easily done by restricting the population on which we standardise to the subset of interest. Once again, we use the same population to obtain estimates under treatment and under no treatment. However, this time only the covariate distribution of a specific subset is used for the standardisation. For instance, by applying the empirical covariate distribution of breast cancer patients who received hormonal therapy (i.e. restricting our estimates only to those who received hormonal treatment), the 10-year standardised relapse-free survival probability is equal to 0.32 (95% CI: 0.28–0.37) under hormonal therapy and 0.24 (95% CI: 0.22–0.26) under no hormonal therapy. This results in a difference in 10-year standardised survival probabilities of 0.08 (95% CI: 0.04–0.13) within the treated group (Fig. 6). The standardised survival probabilities within the treated group is lower than the survival within the total population (Fig. 3). This is expected as the patients who had hormonal therapy were older, had a higher number of positive nodes, and there was a larger proportion of tumours above 50 mm in comparison to patients who did not receive hormonal therapy (Table 1).

## Discussion

When estimating the effect of a treatment (or exposure) on a time-to-event outcome, it is important to consider potential imbalances in the groups of comparison and as far as possible adjust the statistical analysis for confounding variables. This can be done by fitting regression models. In this article, we have reviewed different ways to do this and provided illustrative examples of how regression models can be depicted graphically. We showed that absolute values for differences in standardised survival can be obtained by estimating standardised survival probabilities for each treatment arm. The association between treatment and the outcome of interest can then be quantified by calculating the difference in standardised survival probabilities by treatment status.

Currently, HRs are commonly reported as the main measure after fitting regression models to time-to-event data, i.e. a relative rather than absolute value for the effect of treatment (or exposure). In comparison, the difference in standardised survival probabilities provide an estimate of the absolute risk with/without treatment (or exposure) and are thus in general more informative than the HR. Comparing the effect of treatment on survival using absolute rather than relative measures makes it easier to understand whether treatment results in clinically meaningful improvements in survival. Moreover, survival probabilities have a less challenging interpretation in comparison to hazard ratios. Often HRs are misinterpreted as relative risks. However, the HR is simply calculated as the ratio of hazard rates and corresponds to relative rates. In contrast, the survival probability estimates the probability of not experiencing the event by a specific time and its complement (1 minus survival probability) can be interpreted as the risk. Further, HR estimates are affected by built-in selection bias, as HRs are conditional on individuals that have not yet experienced the event, which is not the case with survival probabilities [6, 19]. Finally, the effect of treatment is often reported as a single HR for the whole study duration assuming that the treatment effect remains constant during follow-up. For many settings, this is not a realistic assumption and time-dependent HRs can communicate the experience of patients more accurately (e.g., by plotting the time-dependent hazard ratio of age across time since diagnosis). The difference in standardised survival probabilities provides a summary of the treatment effect using a single measure for each time of interest even after fitting complex models with various time-dependent effects and interactions. If the covariates we have adjusted for in the analysis are sufficient for confounding control, then the difference in standardised survival probabilities can be interpreted as the population causal effect [11]. It is important to note, though that this interpretation relies on the validity of the unmeasured confounding assumption (which is based on subject matter knowledge) and requires careful consideration, as often it is not possible to adjust for all relevant confounders. Adjusting for sufficient confounders is important also when hazard ratios are of interest and is not specific only to standardised survival curves. In practice, sensitivity analyses to modelling assumption are recommend as a way of assessing their impact on the estimates of interest. Moreover, in this paper, we focus on examples with no competing events, however, the interpretation of the standardised survival probabilities is less straightforward in the presence of competing events. If the competing events are conditionally independent, standardised cause-specific survival curves can be obtained after fitting cause-specific regression models. Otherwise, standardised cumulative incidence functions for the event of interest in the presence of competing events can be obtained instead, and these are discussed in detail elsewhere [22].

Compared to adjusted survival curves using the mean covariate method, standardised survival probabilities are obtained by averaging the conditional survival estimates of all individuals in the study population. In this way, the empirical covariate distribution is applied and the standardised estimates correspond to the estimates for the observed covariate distribution in the overall population instead of setting the adjusting covariates to a fixed and potentially meaningless value (e.g., mean observed value), as when obtaining “naively” adjusted survival curves. Standardising to a subset of the population can also be done by restricting the estimates to a particular subset (e.g. treated) and this help us address important clinical questions regarding the potential impact of interventions on individuals who did not receive the intervention yet or the impact of an intervention on individuals who actually received the intervention. Standardised survival estimates, as well as confidence intervals, can easily be obtained using standard existing software. A code example is provided in the supplementary material, with the standard errors obtained using the delta-method [23, 24]. In our analysis, we fitted a flexible parametric survival model that can incorporate complex effects easily. However, standardised survival curves can, in principle, be obtained after fitting any survival model and have also been implemented in R for the Cox model [11]. Finally, even though in this paper our focus was on using regression standardisation to obtain standardised survival probabilities, marginal probabilities can also be estimated using other approaches not presented in the paper, such as inverse probability weights [25]. In the inverse probability weighting approach, instead of adjusting for covariates in the survival model, each individual is given a weight based on the probability of receiving their own treatment conditional on their observed covariate pattern. These weights are obtained from fitting a regression model with the treatment as the outcome, for example using logistic regression for a binary treatment.

Our illustrative example included only baselines covariates (i.e. covariates measured at start of follow-up). However, time-varying covariates may be of interest. In principle, standardised survival curves can also be obtained when the treatment under study is time-varying by estimating the survival curve under a scenario were individuals are “always treated” and the scenario “never treated”. However, careful consideration is required to determine whether this comparison is relevant for the question under study. Obtaining standardised curves is though more straightforward when other covariates included in the model are time-varying. This is because when comparing survival curves under different treatment arms, we only need to set the other adjusting covariates to the same values for both estimated curves to compare the same population. However, the interpretation is in terms of the covariate distribution at the start of follow-up/baseline.

The difference in standardised survival probabilities under different treatment arms is a valuable and informative measure to summarise the effect of treatment while adjusting for several confounding variables. Its estimation is no more complex than frequently reported measures, such as HRs, and so we highly encourage its use as an additional measure for reporting the results of a time-to-event analysis.

## Supplementary information

Supplemental Code

Supplemental Table S1

## Data Availability

The authors use publicly available data on breast cancer patients to demonstrate the different measures. Data can be downloaded from http://www.stata-press.com/data/fpsaus.html. The authors also provide Stata code in the supplementary material.
