# Supplementary material for: Standardised survival probabilities: a useful and informative tool for reporting regression models for survival data
Source: Br J Cancer. 2022 Sep 1;127(10):1808–15. doi: 10.1038/s41416-022-01949-6 (PMC9643385; doi:10.1038/s41416-022-01949-6)
Supplement: Supplementary file 2 — Supplemental Table S1 [file 41416_2022_1949_MOESM2_ESM.docx]

Table S1: Standardised survival probabilities for the event of relapse-free survival by treatment group at specific timepoints and the difference in standardised survival probabilities for the event of relapse-free survival under hormonal therapy and under no hormonal therapy, with 95% confidence intervals.

| Years since surgery | Standardised survival probability | | Difference |
| --- | --- | --- | --- |
|  | Under hormonal  treatment | Under no hormonal  treatment |  |
| 1 | 0.92 (0.91-0.94) | 0.90 (0.89-0.91) | 0.02 (0.01-0.03) |
| 5 | 0.63 (0.60-0.67) | 0.56 (0.54-0.58) | 0.07 (0.04-0.11) |
| 10 | 0.48 (0.43-0.53) | 0.39 (0.37-0.41) | 0.09 (0.04-0.14) |
